# Vascular Endothelial Growth Factor (VEGF) Serological and Lacrimal Signaling in Patients Affected by Vernal Keratoconjunctivitis (VKC)

**DOI:** 10.1155/2018/3850172

**Published:** 2018-09-16

**Authors:** Marcella Nebbioso, Andrea Iannaccone, Marzia Duse, Michele Aventaggiato, Alice Bruscolini, Anna Maria Zicari

**Affiliations:** ^1^Department of Sense Organs, Sapienza University of Rome, p. le A. Moro 5, 00185 Rome, Italy; ^2^Department of Pediatrics, Sapienza University of Rome, p. le A. Moro 5, 00185 Rome, Italy; ^3^Department of Experimental Medicine, Sapienza University of Rome, p. le A. Moro 5, 00185 Rome, Italy

## Abstract

**Background:**

Vernal keratoconjunctivitis (VKC) is a rare inflammatory disease involving the ocular surface, with seasonally exacerbated symptoms. Both type-1 and type-4 hypersensitivity reactions play a role in the development of VKC.

**Purpose:**

The aim of the present study was to assess the presence and evaluate the concentration of the vascular endothelial growth factor (VEGF) in tear and blood samples from patients with VKC, during the acute phase, based on the histopathological vasculostromal structure of the tarsal papillae.

**Methods:**

Two groups of children aged between 6 and 16 years of life were enrolled: 21 patients (16 males, 76%) affected by VKC, tarsal or mixed form, and 13 healthy children (5 males, 38%) used as controls. Blood and tear samples were obtained from all patients, in order to specifically assess the presence of VEGF. Statistical analyses were performed with one-way ANOVA, followed by post hoc comparisons with the Bonferroni tests. Pearson's correlation was chosen as statistical analysis to assess the relationship between the expression levels of VEGF in tears and blood and the clinical parameters measured.

**Results:**

Comparing the 2 groups for VEGF concentration, a statistically significant difference was found in tear samples: the mean value was 12.13 pg/mL (±5.54 SD) in the patient group and 7 pg/ml (±4.76 SD) in controls (*p* < 0.05). However, no statistically significant difference was found when comparing VEGF concentration in blood samples (*p* > 0.05), with a mean value of 45.17 pg/mL (±18.67 SD) in VKC patients and 38.08 pg/mL (±19.43 SD) in controls.

**Conclusions:**

This pilot study highlights the importance of lacrimal and vascular inflammatory biomarkers that can be detected in VKC patients during the acute phase, but not in healthy children. The small group of patients warrants additional studies on a larger sample, not only to further investigate the role of VEGF but also to evaluate the angiogenic biomarkers before and after topical treatment.

## 1. Introduction

Vernal keratoconjunctivitis (VKC) is a rare (<1 : 10.000), chronic, bilateral, at times asymmetrical, often severe inflammation of the ocular surface. It was first described in the ophthalmic literature as conjunctiva lymphatica more than 150 years ago. Subsequently, numerous authors referred to it using different names, each one labeling one of the various aspects of the disease [1–3]. VKC especially affects young males, with an average age of onset of 6-7 years and a reported male-to-female ratio varying from 4 : 1 to 2 : 1, and tends to regress during or soon after puberty, disappearing in the majority of cases around 4–10 years after onset. It is more common in dry and hot climates, such as the Mediterranean area, Central Africa, West Africa, the Middle East, Japan, the Indian subcontinent, and South America [3–6]. VKC is typically characterized by seasonal exacerbations, although the most severe forms can suffer from perennial symptoms, and visual impairment may occur if the cornea is involved. According to the predominant conjunctival district involved, three forms of vernal keratoconjunctivitis have been identified and described: tarsal (palpebral), limbal (bulbar), and mixed type. In the first form, more prevalent in Europe and the Americas, large papillae (>1 mm) occur predominantly at the upper tarsus and those with a size of 7-8 mm are known as cobblestone papillae, constituting from the vasculostromal structure of collagen types I and II and proteoglycans [3–5]. The limbal form, which is more seen in Africa, is usually accompanied by limbal infiltrates and Horner–Trantas nodules; the mixed type presents intermediate characteristics between the two. A recent study has suggested an update to this classification that should include a phenotype with a predominant corneal involvement. Even though this was associated with nonspecific alterations of the corneal epithelium, relevant changes were measured by confocal microscopy and they were correlated with the degree of photophobia reported by patients. Corneal changes include punctate epithelial keratitis, epithelial macroerosions, gelatinous limbal hypertrophy, and plaque formation [3–5]. If untreated, the initial damage may lead to the onset of oval-shaped epithelial defects, known as shield ulcers, which are reported to occur in 3–11% of patients [3–6]. These undoubtedly represent the most severe signs of the disease, as they can have a heavy impact on the quality of life and even cause visual impairment or lead to astigmatism, keratoconus, corneal perforations, and scars. Although the allergic nature of VKC has been accepted for a long time, its exact aetiology and pathogenesis are still unclear. The role of hormonal factors is suggested by positive staining for oestrogen and progesterone receptors in the conjunctiva from patients, predilection for male sex, and resolution after puberty [3, 6, 7]. Genetic predisposition also contributes to the development of this disease, and although no relationship has been found yet between VKC and a particular genotype, the constant and increased presence of eosinophils in blood, tears, and conjunctival scrapings, the expression of a multitude of mediators and cytokines, and the predominance of CD4 cells locally suggest that VKC may be a phenotypic model of upregulation of the cytokine gene cluster on chromosome 5q [7–12]. In addition, multiple studies have investigated the family history of allergic diseases (asthma, rhinitis, eczema, urticaria, and dermatitis) and immunological diseases (Hashimoto's thyroiditis, type I diabetes, psoriasis, rheumatoid arthritis, and systemic lupus erythematosus), reporting such an association in roughly half of the patients diagnosed with VKC [3, 5, 6, 13, 14]. At this point, the accumulation of a large amount of immunological data has established that the pathogenesis of VKC is much more complex than a mere type 1 hypersensitivity reaction, also involving IgE-independent (type IV) mechanisms and cytokines, such as the interleukin 17 (IL-17) [7, 10, 12].

The purpose of the present study was to assess the presence and evaluate the concentration of vascular endothelial growth factor (VEGF) in tear and blood samples of patients affected by VKC, during its acute phase, comparing the results with a control group, in order to evaluate the role of VEGF in the pathogenesis of this disease.

## 2. Patients and Methods

The study was performed at the Department of Sense Organs of the “Policlinico Umberto I” and at the Department of Pediatrics, Division of Allergy and Immunology, Sapienza University of Rome. In accordance with the Helsinki Declaration, all parents were informed about the use of their data and informed consent was obtained. The study also fully obeyed the Good Clinical Practice guidelines and was approved by the Ethical Committee of our hospital, Sapienza University of Rome (authorization Rif. CE: 4708, 9/11/2017; Prot. Cod.: VKC06/2017).

**Figure 1:**
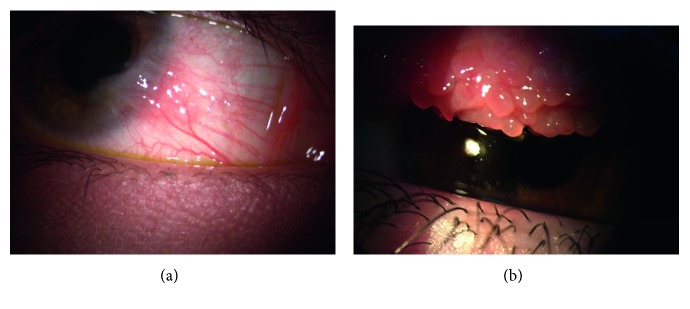
Patient affected from vernal keratoconjunctivitis (VKC). (a) Mixed form with cornel signs limbar neovascular and (b) tarsal papillae on the superior conjunctiva.

We enrolled 21 children (16 males, 76%), aged between 6 and 16 years of life, affected by tarsal and mixed forms of VKC (Figure 1; Table 1). Thirteen healthy children (8 males, 62%) with negative skin prick test (SPT), without allergic, ocular, and systemic disease, cross-matched for sex and age with patients affected by VKC, were used as controls. At the time of enrolment, a complete hematologic and cardiologic examination was performed, systemic blood pressure was determined, and blood samples were collected. A complete ophthalmic evaluation was performed on both eyes through a detailed medical and ocular history. The eye examination included the following:

- Best-corrected visual acuity (BCVA) for far and near distance
- Slit-lamp biomicroscopy
- Intraocular pressure measurements
- Dilated fundus examination

The diagnosis and follow-up were performed by an expert ophthalmologist through a score based on ocular signs such as little and/or giant papillae, conjunctival hyperemia, keratitis, and Horner–Trantas dots and on subjective ocular symptoms such as itching, photophobia, foreign body sensation, tearing, and mucous secretion according to the system described by each variable. In this score, each variable was graded as follows: 0 = absent; 1 = light; 2 = mild; 3 = moderate; and 4 = severe [3, 9]. Patients with a total score ≥10 were included in the study. Some authors subsequently expanded it by including two supplemental parameters: the duration of symptoms (0 if  <1 year, 1 if  >1 year, and 2 if  >2 years) and the worsening of symptoms in spring and summer (0 if not present and 1 if present). Conjunctival scraping was also performed, in order to further improve the diagnosis in patients with VKC. Skin prick tests (SPTs) were performed on each patient for common inhalants and food allergens (Lofarma, Milan, Italy): *Dermatophagoides pteronyssinus* (Der P), *Dermatophagoides farinae* (Der F), dog/cat dander, *Olea europaea*, *Lolium perenne*, *Alternaria alternata*, *Parietaria officinalis*, lactalbumin, *β*-lactoglobulin, casein, egg white and yolk, soy, and cod fish. A positive SPT was defined by the presence of a wheal more than 3 mm respect to the wheal size of the control (saline solution). Serum was obtained from the peripheral blood samples collected from all the children included in the study to evaluate the serum level of VEGF, autoimmunity, ANA, and total IgE (Table 1). Blood samples were obtained at enrolment. In both patients and controls, tear samples were obtained as follows: 20–50 *µ*l of open eye tears was gently collected from the external canthus of the most affected eye using a capillary micropipette and avoiding the tear reflex as much as possible. The samples were placed in Eppendorf Tubes, centrifuged at 160 ×*g* for 8 min, and stored at −80°C. The samples were successively probed using three microwell plate arrays.

## 3. VEGF Level Measurement

Lacrimal and serum VEGF levels in healthy and VKC patients were determined with the VEGF EIA Kit from Enzo Life Sciences and following manufacturer's instructions. Briefly, 100 ml of samples was mixed with 100 ml of the assay buffer and placed in each well of a 96-well plate. The mix was then incubated for 18 h at 4°C. The following day, each well was rinsed with a washing buffer before adding 100 ml of an anti-VEGF antibody/well. The antibody was incubated for 30 min at 4°C. Afterward, 100 ml of a substrate solution was added and incubated for 30 minutes at room temperature (RT) in the dark. After adding a stop solution, the optical density at 450 nm was measured using the GloMax-Multi Detection System (Promega, Milan, Italy). The fluorescence intensity of the assay buffer was subtracted from each experimental sample.

## 4. Statistical Analysis

Data obtained were used for statistical analysis. Quantitative variables were summarized with mean and standard deviation (SD). The differences between groups were evaluated by Fisher's chi-square test or test. The correlations between clinical, demographic, and biological parameters were performed with Spearman's rho. The results are expressed as means ± SD and 95% confidence intervals (95% CIs). Statistical analyses were performed with one-way ANOVA, followed by post hoc comparisons with the Bonferroni tests. Calculation of Pearson's correlation was also chosen as statistical analysis. Values were relieved to assess the relationship between the expression levels of VEGF in tears and blood and the clinical parameters measured. A *p* value < 0.05 was considered statistically significant. Statistical analysis was conducted through the SPSS 18.0 software (Statistical Package of Social Sciences, Chicago, IL, USA).

## 5. Results

**Figure 2:**
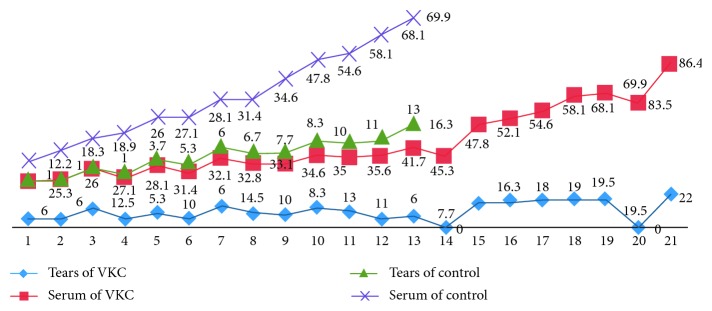
EIA values for VEGF concentration in tears and blood in control and vernal keratoconjunctivitis (VKC) groups.

The study included 21 patients (16 males and 5 females) aged between 6 and 16 years of life (mean 8.62; SD ± 2.65), affected by tarsal and mixed forms of VKC, and thirteen healthy children (8 males and 5 females) aged between 6 and 15 years (mean 7.98; SD ± 3.2). The 2 groups for mean age and gender were not statistically significant. According to literature, VKC prevalence was higher in males than in females (76% vs 24%) in the population we studied [1, 3, 14, 15]. Allergological, serological, and lacrimal tests were normal in the control group, while 11 VKC patients (52.4%) showed positive Skin Prick Test (SPT) for several common inhalants and food allergens. Fifty-seven percent of patients were familiar with allergic diseases and 14% had a parent with autoimmune disease. Comparing the 2 groups after using the VEGF EIA kit for VEGF concentration, a statistically significant difference was found in tear samples: the mean value was 12.13 pg/mL (±5.54 SD) in the patients group and 7 pg/ml (±4.76 SD) in controls (*p* < 0.05) (Figure 2). However, no statistically significant difference was found when comparing VEGF concentration in blood samples (*p* > 0.05), with a mean value of 45.17 pg/mL (±18.67 SD) in VKC patients and 38.08 pg/ml (±19.43 SD) in controls (*p*=0.29) (Table 2).

The correlation of Pearson revealed an *r* value of −0.14, −0.18, −0.15, and −0.12 between the expression levels of VEGF in tears and the clinical parameters giant papillae, foreign body, conjunctival hyperemia, and itching, respectively. Moreover, the correlation of Pearson showed an *r* value of −0.28, 0.01, 0.04, and −0.03 between the expression levels of VEGF in serum and the clinical parameters giant papillae, foreign body, conjunctival hyperemia, and itching, respectively. There were significant correlations between the mean values of VEGF tears and the clinical parameters (*p* < 0.05). However, the statistically significant correlation was only identified between VEGF serum and giant papillae, and there was no relationship between VEGF serum and the other clinical parameters (Table 3).

## 6. Discussion

The purpose of this study was to assess the presence and evaluate the concentration of VEGF in tear and blood samples of patients affected by VKC in acute phase in comparison with the control group in order to assess the pathogenic role of VEGF.

VKC is a rare, chronic ocular disease that affects children, mainly involving males over females, and usually regressing during or soon after puberty. This aspect emphasizes the role of hormonal factors in the pathogenesis of the disease, as pointed by several studies [3, 9, 15]. Although VKC has been always regarded as an IgE-mediated disease, the literature reports that roughly 50% of patients have a positive SPT or high levels of serum IgE. Our data were in line with these findings. Thus, the IgE-mast-cell-mediated process and Th1 response are not enough to explain all the mechanisms of the disease. The role of Th2 responses in VKC has been studied for a long time, and VKC is actually not regarded just as an ocular disease but as a systemic disease with a T-cell-activated response. Many studies underlined the role of single cytokines and mediators in the development of the disease, such as the IL-17 and the HMGB-1 [11–13, 15]. In the present study, we focused on the assessment and quantification of the VEGF, comparing the concentration of such a molecule in tear and blood samples from VKC and control subjects. Tear samples are relatively easy to collect from the patients as a noninvasive diagnostic procedure that provides valuable information on differential tear cytology and levels of mediators at the site of inflammation [7, 10, 11]. The critical limitation of such a procedure is the volume of the tear sample, which ideally should be collected without stimulating the tear reflex, and the quantity of the sample obtainable, often very limited. Determination of tear mediator, cytokine, or chemokine levels is not yet used for diagnosis, but only for the study of allergic physiopathology or for the evaluation of efficacy of antiallergic agents [4, 7, 10, 11, 16]. The concentration and distribution of inflammatory mediators or inhibitors in the tear fluid have been extensively studied in ocular allergy, in an attempt to find a disease marker, to better understand the immune mechanisms involved in the ocular surface inflammation and to identify potential targets for therapeutic interventions [7, 8].

Epithelial cells, fibroblasts, and inflammatory cells including eosinophils and monocytes/macrophages are possible sources of VEGF in VKC [7, 17]. Angiogenic and growth factor pathological expression is frequently associated with the expression of matrix metalloproteases (MMPs) [7]. MMPs cause the extravasation of leukocytes through limited proteolysis of basement membranes; degradation of proteoglycans, laminin, fibronectin, and collagens; and activation of the precursor forms of other MMPs. An imbalance between MMPs and their natural tissue inhibitors (TIMP) is also probably involved in the pathogenesis of conjunctival inflammation, remodeling, and corneal changes in VKC. In fact, MMP-1, MMP-2, MMP-3, MMP-8, MMP-9, and MMP-10 are highly expressed in all forms of active VKC, consequently to the increase of proinflammatory cytokines, such as IL-1b and tumor necrosis factor-α (TNF-*α*) [7]. Moreover, chronic conjunctival inflammation in VKC is associated with increased expression of α3 and α6 integrin subunits' receptors, epidermal growth factor receptor (EGFR), VEGF, transforming growth factor-b (TGF-b), basic fibroblast growth factor (bFGF), and platelet-derived growth factor (PDGF), thus suggesting a possible contribution of integrins, EGFR, and growth factors in VKC conjunctival remodeling and also a role of growth factors in the expression and function of integrins [8].

On the other hand, an anti-VEGF therapy could be helpful for anterior segment eye disease as an adjunct in the treatment of allergic and immunologic eye diseases. The most important growth factor in corneal angiogenesis is VEGF, and it is upregulated during corneal neovascularization (NV). Recently, anti-VEGF therapies are one of the most important drugs used for corneal NV treatment. In fact, anti-VEGF therapies (bevacizumab, ranibizumab, and aflibercept) have shown efficacy in attenuation of corneal neovascularization in both animal models and clinical trials [22, 23]. Case reports in this area include, for example, topical bevacizumab use in a patient with long-standing ocular cicatricial pemphigoid unresponsive to steroid, but moderately responding to the treatment, a single case of ocular graft-versus-host disease that responded partially to subconjunctival bevacizumab, and a report of topical bevacizumab application in 2 patients with Stevens–Johnson syndrome [22, 23]. Visual acuity improved in all 3 eyes, with decreased corneal NV, haze, and conjunctival injection without any serious adverse events. In conclusion, bevacizumab has partially reduced corneal NV through different routes of administrations: topical, subconjunctival, and intraocular application, while early treatment with subconjunctival administration of ranibizumab may successfully reduce corneal NV. Establishment of safe doses is highly important before these drugs can be involved in the clinical setting [22, 23].

Thus, VEGF becomes a potential marker for the disease and/or its severity. It is our aim to cover in a future study how the tear levels of such a mediator could possibly be modified before and after treatment with therapeutic ocular drops [4, 18–21]. When evaluating blood samples, the difference was not statistically significant. However, we found a statistically significant difference of the VEGF concentration in tears between VKC and control groups. Again, this finding suggests the need for further studies, which should include a larger number of patients suffering from VKC. Then, VEGF concentration could be evaluated before and after treatment with cyclosporine or anti-VEGF ocular drops to investigation to better understand its role in the pathogenesis of the disease. In summary, it might even be seen as a potential therapeutic target.

## Figures and Tables

**Table 1:** Demographics and clinical grading of 21 vernal keratoconjunctivitis (VKC) patients.

Characteristics of VKC patients		
Sex	16 males (76%)	5 females (24%)
Age (from 6 to 16 years)	Mean 8.62	SD ± 2.65
VA (BCVA)	20/20	SD ± 0.0
Atopic SPT	11 positive (52.4%); major allergens: house dust and grasses	10 negative (47.6%)
Family condition	12 (57%) familiar with allergic DS	3 (14%) familiar with autoimmune DS
VKC forms	Tarsal: 13 (62%)	Mixed: 8 (38%)
Vitamin D levels (VKC vs controls) (in ng/ml)	19.8 ± 6.7 vs 24.7 ± 6.5	Lower levels if compared to controls (*p* < 0.0001)

ANA positivity	30.8%	Total IgE 4100 UI/ml	46.1%
Photophobia (grade 4)	42.3%	Secretion or tearing	28.8%
Foreign body sensation	25.4%	Itching and conjunctival hyperemia	11.5%

SD: standard deviation; VA: visual acuity; BCVA: best-corrected visual acuity for far distance; SPT: skin prick test for several common inhalants and food allergens; DS: diseases.

**Table 2:** Comparisons of the 2 groups after using the VEGF EIA kit for vascular endothelial growth factor (VEGF) concentration.

VKC group (*N*=21)	Control group (*N*=13)	*p* value
VEGF tears in pg/mL: 12.13 ± 5.54	Tears in pg/mL: 7 ± 4.76	<0.05 (0.011)
VEGF serum in pg/mL: 45.17 ± 18.67	Serum in pg/mL: 38.08 ± 19.43	NS > 0.05 (0.29)

SD: standard deviation; NS: not statistically significant. The values are expressed as mean ± SD. A statistically significant difference was found in tear samples (*p* < 0.05). No statistically significant difference was found when comparing VEGF concentration in blood samples (*p* > 0.05).

**Table 3:** Pearson correlation coefficient (CC) results (parametric test) (statistical significance was set at *p* < 0.05).

VKC group (*N*=21), mean value in pg/mL (±SD)				
VEGF relationship		Clinic score	Pearson CC	*p* value
Tears/giant papillae	12.13 (5.54)	3.43 (0.59)	−0.14	<0.05
Tears/foreign body	12.13 (5.54)	3.24 (0.83)	−0.18	<0.05
Tears/conjunctival hyperemia	12.13 (5.54)	2.76 (0.97)	−0.15	<0.05
Tears/itching	12.13 (5.54)	2.57 (0.73)	−0.12	<0.05
Serum/giant papillae	45.17 (18.67)	3.43 (0.59)	−0.28	<0.05
Serum/foreign body	45.17 (18.67)	3.24 (0.83)	0.01	NS
Serum/conjunctival hyperemia	45.17 (18.67)	2.76 (0.97)	0.04	NS
Serum/itching	45.17 (18.67)	2.57 (0.73)	−0.03	NS

NS: not statistically significant.

## Data Availability

The data of this study can be obtained from the medical database of Policlinico Umberto I, Sapienza University of Rome or from the corresponding author upon request.
